# Bridging juvenile justice and behavioral health systems: development of a clinical pathways approach to connect youth at risk for suicidal behavior to care

**DOI:** 10.1186/s40352-021-00164-4

**Published:** 2021-11-29

**Authors:** Gail A. Wasserman, Katherine S. Elkington, Gail Robson, Faye Taxman

**Affiliations:** 1 Department of Psychiatry, College of Physicians and Surgeons Columbia University, New York, USA; 2 Columbia University and New York State Psychiatric Institute, 40 Haven Avenue, Kolb Annex Rm 273, New York, NY 10032 USA; 3 George Mason University, Virginia, USA

**Keywords:** Bridging, Suicidal behavior risk, Juvenile justice, Linkage

## Abstract

**Background:**

Justice-involved youth have high rates of suicidal behavior and co-morbid psychiatric disorders, yet low rates of service use. Implementation efforts aimed at supporting cross-agency linkage protocols may be useful components of interventions promoting behavioral healthcare service access for youths on probation. The purpose of this study was to develop clear referral Pathways for three suicide risk classifications of youth, across 10 counties in a single state through a community-academic partnership in New York state, a strategic planning process between county Probation departments and community Behavioral Health.

**Results:**

We sought to clarify service destinations for youth in three classes of risk for suicidal behavior: Class I (Crisis, Imminent Risk); Class II (Crisis, Non-Imminent Risk); and Class III (Non-Crisis but in Need of Service). Prior to Pathway Meetings, there was a low degree of agreement between Probation and Behavioral Health leadership for the appropriate service destination for youths in crisis, whether at imminent risk (Class I: 57.8% overlap) or at lower than imminent risk (Class II: 45.6% overlap). Options for referral destinations for Classes I and II decreased significantly (indicating greater overlap) as a result of Pathway Meetings [(Class I: from 2.5 to 1.1 (t_(9)_ = 3.28, *p* < 0.01); Class II: from 2.8 to 1.3 (t_(9)_ = 4.025, *p* < 0.003)]. Pathway Meetings allowed Behavioral Health *and* Juvenile Justice systems to make joint decisions regarding referral pathways, resulting in innovative solutions, such as the use of mobile crisis.

**Conclusions:**

The community-academic partnership served to bring internal (Juvenile Justice) and external (Behavioral Health) contexts together to successfully generate agreed upon Pathways to care for youths demonstrating risk for suicidal behavior. Bridging Behavioral Health *and* Juvenile Justice systems together to agree to referral Pathways for each risk class can increase appropriate service use.

**Trial registration:**

ClinicalTrials.gov, NCT03586895. Registered 21 June 2018,  https://register.clinicaltrials.gov/prs/app/template/EditRecord.vm?epmode=Edit&listmode=Edit&uid=U0003B7I&ts=4&sid=S00080NN&cx=-n4kinh

## Background

Among adolescents in the US, suicide is the second leading cause of death (Ivey-Stephenson et al., 2020), with rates increasing 24% between 1999 and 2014 (Ruch et al., 2019). Compared to youth in the general population, those involved in the juvenile justice system are at even greater risk for suicidal behavior, given their increased prevalence of mood and substance use disorders, trauma exposure and access to firearms (Wasserman et al., 2009). Although great strides have been made in screening and service provision for the nearly 50,000 youth in secure juvenile justice settings, practices and programs aimed at identifying and addressing suicidal behavior are rare for the nearly 400,000 youth who are supervised while residing in their home communities (e.g., in Probation settings: Wasserman et al., 2009). For justice-involved youths remaining in their home communities, service needs are usually identified in one service sector (the juvenile justice system), while treatment is provided in another (typically the Behavioral Healthcare system), complicating the transition to care (Wasserman et al., 2021). Further increasing the difficulty in these transitions, youths in contact with the juvenile justice system often share an array of social and family risks, including antisocial attitudes or peers, and fewer family supports (White et al., 2016) that make navigation between service systems even more challenging.

Lack of inter-agency service co-ordination can also create barriers to service access. In a recent multistate examination of over 8000 youths undergoing intake into community juvenile justice agencies (Wasserman et al., 2021), over 75% were screened for behavioral health concerns, and over half of those were identified as requiring further evaluation or treatment. Yet, only one in five “identified” juveniles was referred on for further clinical assessment or treatment and only about two thirds of those referred actually initiated treatment. While that study did not examine risk for suicidal behavior specifically, the low referral rate for the wider range of behavioral health disorders in general that were examined illustrates failures in current practice. Service transitions for those at risk for suicidal behavior (SB) would require particular attention to inter-agency communication and coordination to facilitate access to and retention in care, while poorly developed or absent referral and linkage procedures and limited interagency collaboration increase the likelihood that these youths fail to make the transition across systems (Waserman et al., 2009, 2021).

The work described here reflects foundation-building efforts for a multisite intervention (*e-Connect)*, based in New York State. e-Connect is a systems-level program for identifying risk of SB in juvenile probationers and supports their cross-system linkage to community behavioral health providers (R01MH113599). e-Connect trains POs, formalizes interagency collaboration, and uses a web-based digital clinical decision support application to seamlessly combine (a) evidence-based **screening** for SB and related behavioral health (BH) problems, (b) classification of clinical **need,** and (c) county-specific streamlined **referral** plans for Behavioral Health services. This manuscript describes our efforts to generate those county-specific referral plans and facilitate inter-agency service coordination by guiding consensus-building efforts between local probation and behavioral health agencies.

### Theoretical framework and project background

A common implementation science framework, the Exploration, Preparation, Implementation, Sustainment (EPIS) framework, acknowledges the importance of two areas of focus, “inner” (the agency or service system in which practice change is planned) and “outer” (other agencies/systems in the interagency network) contexts, for the successful delivery of evidence-based practices (EBPs). The interplay between inner and outer contexts helps to identify barriers and facilitators to the implementation and sustainment of EBPs. Recently, the EPIS developers added *bridging factors* to this framework to highlight the importance of critical interactions and processes within interorganizational systems (Lengnick-Hall et al., 2020; Moullin et al., 2019). Bridging factors “cross or link the outer system with the inner organizational context,” (Moullin et al., 2019) and include relational factors (e.g., community-academic partnerships), role clarifications (e.g. boundary spanning individuals or organizations that are able to close structural holes between groups, such as facilitators), and formal arrangements (e.g., contracts or policies, such as data sharing agreements).

Relatively little attention has focused on bridging factors and how they affect operations. In a recent review of studies employing the EPIS model, just under a third described bridging factors, compared to 90% and 57% reporting on the inner and outer context, respectively (Moullin et al., 2019). Most examinations of bridging factors focus on contractual relationships, but some have illustrated how operational features such as shared assessments, modifications to program procedures, sharing budgets, joint case management practices, and other joint policies help to facilitate interagency collaboration (Taxman & Bouffard, 2000; Fletcher et al., 2009; Lehman et al., 2009).

Community-academic partnerships represent a particular type of bridging factor that serves to connect inner and outer contexts to achieve a shared goal. Academic partnerships are usually relationship-focused and support the addressing of especially hard-to-navigate issues (Rudes et al., 2014). This may be because such partnerships offer longer term relationships, in which the academic partner is more familiar with the historical factors related to the issues under scrutiny. This deeper understanding allows the academic partner to provide support to address the socio-political factors that might otherwise stall progress on sensitive concerns (Rudes et al., 2014).

In the initial stages of foundation-building work for *e-Connect*, clinical researchers at Columbia university and colleagues operated as academic partners, building upon our prior experience (Wasserman et al., 2008, 2009) as clinical services researchers, guiding local agencies in assessing the behavioral health concerns of youth in juvenile justice settings and then linking them to care (Wasserman et al., 2008, 2009; Elkington et al., 2020). Our immediate goal was to generate Pathways to care for youths at various levels of risk that would be clinically sound, well-defined, straightforward, and mutually agreed-upon.

The strategic planning process for developing this linkage framework involved querying Juvenile Justice and Behavioral Health leadership from 10 participating counties about their existing Juvenile Justice and Behavioral Health coordination and service capacity, and then conducting onsite meetings to identify Pathways to care attuned to youths’ presenting clinical risk level and the array of local county resources. Each county established a change team with representatives from its Juvenile Probation and Behavioral Health agencies. In most NY counties, a single individual oversees an agency supervising all Behavioral Health (mental health and substance use) services, as well as those for developmental disabilities. In addition, clients seeking local substance use services are often first directed to a county-affiliated Behavioral Health clinic for evaluation. For this reason, and because our focus is on suicidal risk, we refer to the individual overseeing this county agency as the *Behavioral Health Commissioner.* Here, we describe the interagency intervention that consists of strategic planning meetings. This exploration provides the opportunity to both guide and examine the development of care protocols to link youths manifesting risk for suicidal behavior identified in one service sector (inner context; juvenile justice agency) toward linkage to Behavioral Health treatment via interagency collaboration with another service sector (outer context: Behavioral Health system).

## Methods

Key features of strategic intervention planning to craft sound Pathways to care included reliance on an academic-practitioner partnership, a standard set of activities before the Pathway Meetings, the Pathway Meetings themselves, and a standard set of Pathways constructed around the same model. Below, we describe the Pathway Meetings, provide examples of the service pathways that resulted from those meetings, and the ways in which they generated more efficient, and consensually agreed-upon, pathways to care for probationers in crisis.

### Settings

This work took place in ten New York counties. Based on most recent data available (2014–2020), counties reflect a range of rates of adolescent suicide (mean = 9.1 per 100,000 adolescents; range = 1.4–17.6: New York State Department of Health, 2019), annual Probation delinquent intake population (mean = 82 intakes/year; range = 4–245 youths: New York State Division of Criminal Justice Services*,* 2019), NY geographical region, rate of urbanicity (mean = 53% of the county urban; range = 30%- 90%: United States Census Bureau, 2010), and socio-demographic markers (e.g., percent of children below the poverty rate, mean = 19.2%; range = 7.2%–30.5%: American Community Survey, 2019). Counties differed considerably in the relative availability of Behavioral Health service providers (mean = 55 per 100,000 youths; range = 27.5–141) (Centers for Disease Control and Prevention, 2015). Two counties were designated as among those having a shortage of Behavioral Health services by federal standards (United States Department of Health and Human Services, 2020).

### Role of the academic partner

The goal of the academic-community partnership was to bring both Juvenile Justice and Behavioral Health agencies together to ensure collaboration and agreement in developing a clinically sound process for successful linkage of youth at risk for suicidal behavior in the transition from Juvenile Probation to Behavioral Health services. The academic partners’ specific expertise was rooted in our experience as clinical service system researchers, supporting the development of a model for risk classification for youth suicidal behavior and the essential elements of an efficient Pathway to care, and lending credibility to those efforts. Academic partners also served in the role of facilitators, helping to ensure that systems achieved agreement on shared protocols.

#### Risk classification system

Prior to the initiation of the Pathway Meetings, the academic team created a classification system that ranked elements of suicidal behavior to aid agencies in efficient triage of youth. The risk classification protocol provided generic guidance around how and where youth should be referred upon identification of suicidal behavior and related behavioral health needs. To be most useful to settings where clinical support is not readily available (i.e., Probation), it was necessary that the classification structure map onto elements that would be available from youth self-report from agency-conducted behavioral health screening. We began with the classification system used in our earlier model (Project Connect: Wasserman et al., 2009) that had relied on elements similar to those to be utilized in *e-Connect*, albeit in a less integrated or technologically advanced manner. Project Connect was successful in more than doubling the numbers of youth who initiated treatment (Wasserman et al., 2009), although issues around feasibility (e.g. limited integration into practice workflow, cumbersome identification procedures, and complex, non-automated procedures for referral decisions) limited its sustainability in Probation settings. That earlier system considered young persons at three levels of criticality (Emergency, Crisis, and Non-Crisis but in need of services). We compared our original classification to a more recent widely used system (Suicide Prevention Resource Center, n.d.); aside from nomenclature, we found these to be broadly consistent. Two adaptations were made to address the particular needs of justice-involved youths. First, we included consideration of Non-Suicidal Self-Injury to the Class II designation, since there is strong evidence that Non-Suicidal Self-Injury increases risk for future suicide attempts (Nock et al., 2006) and these problems overlap with history of other suicidal behaviors within juvenile justice populations (Wasserman et al., 2010). Second, we further specified the ways in which Substance Use and Mood Disorder, both common in juvenile justice populations (Wasserman et al., 2010), might accelerate other elements of suicide risk which were also reflected in Classes I and II. The final model (Table 1) shows our three risk classes (Class I) Crisis, Imminent Risk; (Class II) Crisis Non-Imminent Risk; and (Class III) Non-Crisis but in Need of Service. Those in Class I included youths reporting recent and acute suicidal behavior; those in Class II reported some combination of less recent/less acute suicidal behavior or other self-harm; those in Class III reported moderate or high symptoms of internalizing or substance use, perhaps coupled with more distant past suicidal behavior.

**Table 1:** *Risk classification system for youth suicidal behavior*

**Class I: Crisis, Imminent Risk**	
• Suicide attempt in the past 4 weeks **OR**	
• Suicidal ideation (in the past week) **AND**	
o a suicidal plan (in the past 4 weeks) **OR**	
o a suicidal attempt (prior to the past 4 weeks)	
**Class II: Crisis, Non-Imminent Risk**	
• Suicidal attempt (in the past year) **AND**	
o high internalizing disorder symptoms (in the past year) **OR**	
o moderate internalizing disorder symptoms (in the past year) AND high substance use disorder symptoms (in the past year) **OR**	
o suicidal ideation (in the past month) **OR**	
• Suicidal ideation (in the past month) **AND**	
o high internalizing disorder symptoms (in the past year) **OR**	
• Suicidal plan (in the past month) **OR**	
• Suicidal ideation (in the past week) **OR**	
• Non-suicidal self-injury (in the past 3 months)	
**Class III: Non-crisis, in need of Behavioral Health services**	
• Suicidal attempt (in the past year or in lifetime) **OR**	
• Suicidal ideation (in the past month)	
a	
• High internalizing disorder symptoms (in the past year) **OR**	
• Moderate internalizing disorder symptoms (in the past year) and high substance use disorder symptoms (in the past year) **OR**	
• High substance use disorder symptoms (in the past year) **OR**	
• Suicidal plan (in the past year) **AND**	
o high internalizing disorder symptoms (in the past year) **OR**	
o moderate internalizing disorder symptoms (in the past year) **AND** high substance use disorder symptoms (in the past year) **OR**	
o suicidal ideation (in the past year)	

#### Pathway framework

Following the development of the classification system, it was essential to map the route to care, which we describe as *Pathways*. To ensure that a youth successfully initiated appropriate treatment, we envisioned that Pathways would be unique to each locale, but that they would be derived from certain consistent underlying principles regarding level of care. First, we reasoned that it was essential to specify the service to which a youth should be referred, based on the level of risk for suicidal behavior identified, as the destination for that class of youths in the service delivery Pathway. Relatedly, we assumed that a county’s reliance on a single, well-defined pathway for youth in crisis (Classes I and II) would be most efficient, speeding and simplifying access to care. Next, we reasoned that the concrete steps describing how to connect that person to needed services should be specified. Finally, because each county’s array of services was likely to be different, our approach meant that both the referral decisions and the steps to get to the best destination should be defined by county Probation and Behavioral Health leadership working together. The external academic partner facilitated the sessions to focus on the Pathway goals, development of common language, and discussion about resources. This process allowed development of well-defined, straightforward service Pathways that were relatively consistent across counties while remaining sensitive to the variability in services.

### Preparing for pathway meetings

Following an initial preparation call with each county’s Probation Director and Behavioral Health Commissioner, each completed an online Readiness Worksheet that inquired about how, in their county, youths move between their service systems, what barriers and facilitators impact that movement, how agency leadership thought youth at each level of risk for suicidal behavior should be managed, how their agencies approached cross-agency information sharing, and how they documented their internal processes for intake, referral, and treatment. While there was considerable overlap in versions for the two kinds of agencies, Worksheets also inquired more specifically about operations within that particular agency (so that Probation Directors were asked about barriers to making behavioral health service referrals, while Behavioral Health Commissioners were asked about what works well within their agency that supports justice-involved youth’s referral for services or treatment initiation).

This information was integrated into a presentation for each county’s Pathway Meeting that highlighted similarities and differences between agencies in perceptions of their relationship and, in their current linkage activities. This process aggregated responses to provide an accurate picture of those perceptions, with focus on the service delivery features, and minimizing possible negativity in terms of past efforts, in order to develop positive solutions.

A 3-h onsite meeting was held in each county. Meetings included members of the research team, and the local change team, including the Probation Director, the staff member in charge of Juvenile Probation services, and the Behavioral Health Commissioner. Agencies were invited to include other relevant representatives of their leadership teams; Behavioral Health Commissioners generally included staff in charge of local Crisis Services (if the county offered these), and representatives of the most common direct Behavioral Health service provider for youth on Juvenile Probation in that county.

### Conducting pathway meetings

The objectives of the Pathway Meetings were to determine referral processes and the destination settings for youths screened and classified as demonstrating risk of suicidal behavior; to do so, the meetings also arrived at interagency agreement on the steps needed to support service access and information exchange. The Pathway Meetings reviewed what is known about risks for suicidal behavior to ensure that there was consensus about what features should prompt action for justice-involved youths. Similarly, the mapping of the survey findings allowed for identification of points where youths commonly *“*fall through the cracks*”*. The meeting also allowed the academic partner to facilitate discussions about the planned approach to screening and risk classification. If both agencies’ worksheets differed regarding the preferred destination for a particular Class of youths, discussion initially focused on defining the best option(s) and then achieving consensus across agencies. When both partners’ worksheets identified the same destination, this was reinforced. Working backward from the agreed-upon destination, the group was challenged to consider the logical precursor to each step. Next, participants considered contingency planning if the steps on a referral Pathway could not be followed (e.g., family refusal, immediate service unavailability).

All meetings were recorded for the purpose of generating correct Pathways, and transcriptions were reviewed in the subsequent weeks. Recordings were destroyed once de-identified transcripts were generated and Pathways established. Any missing or unresolved information was clarified in subsequent telephone calls. In an iterative process, across counties, steps were placed into a consistent framework that corresponded to the requirements of agency practice (e.g., to provide immediate officer support, Probation Supervisors were first to be notified after screening identified a youth at Class I or II risk) or clinical practice (e.g., the youth, and then the family was informed of screening results; an existing provider, if any, was alerted). Pathway steps were organized into three sets of activities: 1) Debrief (local supervisory staff, youth and family), 2) Connect (youth to agreed-upon service destination), and 3) Document (actions in case records).

### Measures

The study developed surveys and questionnaires for the academic-practitioner partnership.

#### The e-connect readiness worksheet

This worksheet was intended to identify system needs, potential barriers, and current screening and referral protocols as reported by agency leadership. The worksheet was completed prior to the Pathway Meeting. It inquired about participants’ satisfaction and attitudes towards current screening, linkage practices, and where probationers should be referred based on their suicidal behavior risk classification. Separate versions of the worksheet were developed for Probation and Behavioral Health leadership. Probation and Behavioral Health leadership were asked to indicate the provider(s) at which youths identified within each of the three suicidal behavior risk classes should be seen. Responses from both types of agencies were cross-checked against provider websites to identify duplication, and to ensure that a provider designated by slightly different names was indeed a single entity.

### Data analysis

We reasoned that fewer service destinations for a given class of youth represented a more efficient route to care, and that the clarity reflected in fewer service options might increase the likelihood of service access, especially for youth in crisis (Classes I and II). Since Class III included a wide range of disorder types (that might require a range of service destinations, with that number varying across counties), and because Class III covered young persons who were not considered in crisis, we examined efficiency only for those whose screening might place them into Class I or II. As indicators of efficiency, we examined the number of service destinations before (from Worksheets) and after final Pathways were determined.

Worksheet responses from Behavioral Health and Juvenile Justice leadership were independently examined by two reviewers, demonstrating 85% agreement in number and identity of proposed service destinations. We used paired t-tests to compare, for all 10 counties, the **number** of referral destinations from worksheets (pooling destinations offered by either agency) to the number of final service destinations agreed upon at Pathways Meetings. To determine percent **overlap** in proposed service destinations between Probation and Behavioral Health agencies, we divided the number of destinations determined for each Class by the number of unique destinations cumulatively offered by both agencies on their Worksheets. As an example, if the Juvenile Justice Worksheet listed two service destinations and the Behavioral Health agency Worksheet noted three destinations, only one of which was also on the Juvenile Justice agency list, we coded this as reflecting one overlapping suggestion out of four offered destinations, or 25%. By design, overlap in service destinations resulting from Pathway Meetings was 100%.

## Results

### Changes in service destination options before and after pathway meetings

A single county Behavioral Health agency reported an existing formal agreement between Probation and Behavioral Health provider agencies, although the partner Probation agency denied knowledge of such an agreement. Prior to the Pathway Meeting, approximately 60% (57.8%) of the suggested Class I destinations overlapped across agencies, while fewer than half (45.6%) of the preferred destinations overlapped for Class II youth. By design, overlap in service destinations for these Classes was complete after the Pathway Meetings. As Table 2 shows, the average number of options for referral destinations for Classes I and II offered by either Behavioral Health or Juvenile Justice agency leadership decreased (in the direction of more efficiency) significantly after the Pathway Meetings, and they did so in most counties. For Class I destinations, options decreased on average from 2.5 to 1.1 (t_(9)_ = 3.28, *p* < 0.01); for Class II destinations, options decreased from 2.8 to 1.3 (t_(9)_ = 4.03, *p* < 0.003).

**Table 2:** *Changes in Number of Provider destinations before and after Pathway Meetings*

	Pre-Pathway Meeting Service destinations		Final Service destinations	
	Mean	SD	Mean	SD
Class I	2.5	1.3	1.1	0.3
Class II	2.8	1.1	1.3	0.5

### Post-pathway meeting standard pathways

Pathways were to be used by Probation Officers as a guide to link youths, based on identified risk of suicidal behavior during the intake process and agreed-upon local services. Table 3 presents generic Pathways developed for all three suicidal behavior risk classes. Overall, the county Pathways were broadly similar, differing primarily in which local crisis service provider (i.e., Mobile Crisis Team, local law enforcement) would be called upon to address the suicidal behavior risks and in the agreed upon service destination for that Class. The local Class I crisis provider was usually a Mobile Crisis Team (*n* = 7 counties) or local law enforcement (*n* = 3).

**Table 3:** *Standard Pathways, Classes I, II and III*

CLASS I: CRISIS, IMMINENT RISK DO NOT LEAVE YOUTH UNATTENDED	
**DEBRIEF**	
1. Alert supervisor	
2. Inform youth, and then caregiver, of the screening result and next steps	
**CONNECT**	
3. Call **[Class I crisis provider]** and advise them of a Class I/suicidal youth	
4. Distribute the copies of the e-Connect Provider Referral Form and Bidirectional Release Form to the **[CLASS I crisis provider];** keep one copy of each in youth’s case file	
5. If **[CLASS I crisis provider]** determines youth needs to be transported for emergency services, complete and give caregiver the e-Connect Emergency Services/Transportation Form and Home Safety Information Sheet	
**DOCUMENT**	
6. Update supervisor	
7. If youth is already in treatment, inform the existing provider of the e-Connect screening result and events	
8. Document into Caseload Explorer	
CLASS II: CRISIS, NON-IMMINENT RISK DO NOT LEAVE YOUTH UNATTENDED	
**DEBRIEF**	
1. Alert supervisor	
2. Inform youth, and then caregiver, of the screening result and next steps	
**CONNECT**	
3. Call to [**wording about Class II appointment within 72 h**]	
4. **[Step about alerting other providers/LE etc if necessary]**	
5. **[Email/Fax]** e-Connect Provider Referral Form and Bi-Directional Release Form to **[PROVIDER**]; keep one copy of each in youth’s case file	
6. Complete and give caregiver the e-Connect Family Appointment Form and Home Safety Information Sheet	
**DOCUMENT**	
7. Update supervisor	
8. If youth is already in treatment, inform existing provider of the screening result and events	
9. Document into Caseload Explorer	
**CONNECT**	
10. Follow-up with provider immediately after the scheduled appointment time to verify youth’s attendance	
**DOCUMENT**	
11. Update supervisor	
12. Update Caseload Explorer	
CLASS III: IN NEED OF BEHAVIORAL HEALTH SERVICES	
**DEBRIEF**	
1. Inform youth, and then caregiver, of the screening result and next steps (see back of this page)	
2. If already in treatment with a provider, ask if they would prefer to see the existing provider or a new referral	
**CONNECT**	
3. If same provider, connect the youth with their existing provider; make a follow up appointment from your office, with family present or make a plan with caregiver if appointment cannot be made that day	
4. If a new referral, inform youth and caregiver to **[directions to access providers]**	
5. Complete and give caregiver the e-Connect Family Appointment Form	
6. Once known, email/fax e-Connect Provider Referral Form and Bi-Directional release Form to **[PROVIDER];** keep one copy of each in youth’s case file	
**DOCUMENT**	
7. Document into Caseload Explorer	
8. Verify youth attendance with provider within 72 h of their appointment	
9. Update Caseload Explorer	

In all cases of Class I or II, Pathways included a reminder to the Probation Officer conducting the screening that the youth should not be left alone at any time until the designated crisis responder arrived and took over responsibility. For all Pathways, regardless of Class, the structure included three phases: Debrief, Connect and Document. For the D*ebrief phase*, agencies agreed that the Intake Officer’s supervisor should first be informed, providing backup if needed. Next, the officer would inform the youth about the results of the screening and that the accompanying parent would then be debriefed. Training materials provided scripted materials for the family debriefing. In the *Connect phase*, officers were to contact the designated provider to request that they either provide further assessment (if the crisis provider was a clinician) or transport the youth directly to the designated local provider (if this service was to be undertaken by law enforcement or an ambulance service). Release forms and supportive information sheets were to be made available to the family at this point. Pathway steps reminded officers to remain safely with the youth until Crisis Providers appeared. Finally, during the *Document phase*, officers were to inform the youth’s current behavioral health provider (if any) of screening results and agency actions, and then document agency actions and the outcome of any clinical assessment (if known) in case records.

For youths identified as in Class III, the officer was expected to inform the youth about the results of the screening and the accompanying parent would then be debriefed, relying on training materials. Youths already in treatment were offered a supplemental referral to that provider; if not already in treatment, or if they preferred a new provider, they were offered a new referral. Appointments were made directly with the provider with the family present, or a plan was made with the family for them to set up an appointment later that day. Release forms and supportive information sheets were made available to the family at this point. Finally, in the Document phase, officers were to document their actions and the outcome of any clinical assessment (if known) in case records.

### Variability in crisis responders for class I and II

Although each county reached consensus regarding the types of activities that would need to occur to best ensure linkage to service providers, counties differed in the availability of needed services. As a result, while Pathways are quite consistent, they vary across counties as to the designated crisis responder, and which location(s) were chosen to best promote efficient access. The types of services used in Classes I and II, and how they would be included in the linkage process are shown in Table 4. Note that in one county, Probation services were offered in two settings, one of which was co-located within the county Behavioral Health agency offices. The 10 counties therefore had a total of 11 options based on their unique service delivery features.

**Table 4:** *County Variability in Pathways*

Class I Crisis Responder	Class II Provider
Mobile Crisis dispatched to Probation, evaluates, takes charge of next steps: 6 counties	Mobile Crisis dispatched to Probation, evaluates, takes charge of next steps: 4 counties
Probation calls 911, either law enforcement or ambulance transports to ER/crisis service: 3 counties	Mobile Crisis will conduct an evaluation at home within 72 h: 1 county
Mobile Crisis does telephone evaluation, takes charge of next steps: 1 county	Refer to designated accredited Behavioral Health agency for appointment within scheduled within 72 h: 3 counties
Probation walks youth to collocated MH crisis service down the hall: 1 county	Refer to designated accredited MH agency for appointment within scheduled within 72 h, with alert to Crisis service: 1 county
	Refer to Open Access* if immediately available, if not to designated agency for appointment within 72 h: 1 county
	If in treatment with designated provider, schedule appointment for within 72 h; if not in treatment, accompany to Open Access down the hall: 1 county

*Open Access refers to walk-in clinic times during which an intake can occur without a set appointment time

For Class I youths, for whom immediate clinical assessment was needed, the process varied. In the one county where Probation services were co-located in the same building as Behavioral Health services, the officer would accompany the youth to an immediate Behavioral Health agency intake. For the seven counties that had Mobile Crisis Services, the Mobile Team would either be dispatched directly to the Probation Office (six counties) to perform further assessment or would undertake immediate telephone triage. In the remaining three counties, youth transport to the local Emergency Room would be undertaken either by law enforcement or by an ambulance service.

Options for Class II youths, for whom clinical assessment would need to take place within 72 h (during which the youth would remain with his/her family at home), varied by the county’s service delivery system. Four counties elected to have their Mobile Crisis Team institute the Class I protocol and evaluate immediately in the Probation Office. In one county the Mobile Crisis provider agreed to conduct an evaluation at the youth’s home within 72 h. In four counties, an appointment was scheduled at the designated Behavioral Health provider agency for a visit to take place within the next 72 h; for one of these, an alert was provided to the Crisis Team so that they could follow up rapidly to confirm attendance. One county planned to send Class II youths to their accredited walk-in (“Open Access”) service after calling to confirm immediate availability (or for a scheduled appointment within 72 h if a walk-in was not immediately available). Finally, one county arranged for any youth who was already in treatment at the designated provider agency to have a scheduled appointment for within the next 72 h; if the youth was not in treatment, the officer would accompany the youth to the Open Access service which was located in the same building, down the hall. In all instances, connection was to be confirmed by the Officer soon after the 72 h window had passed.

## Discussion

The process described here successfully brought together leadership from partnering agencies, resulting in the generation of agreed upon Pathways to care for youths demonstrating risk for suicidal behavior. Despite variability in the array of services available and in the availability of Behavioral Health service providers, Pathway frameworks for all counties were roughly comparable in structure, while reflecting differences in county resources and programs.

Pathway Meetings and cross-agency effort to identify service destinations for youths in crisis resulted in greater consensus and efficiency regarding best practices for managing Class I and II youths. Prior to Pathway Meetings and their resulting dialogue, Juvenile Justice and Behavioral Health leadership did not have a high degree of overlap in their service suggestions regarding the appropriate destination for youths in crisis, whether at imminent risk (Class I: 57.8% overlap) or at lower than imminent risk (Class II: 45.6% overlap). In practice, when a youth presented with this level of risk for suicidal behavior, this would mean that 40–50% of the time, the juvenile justice agency might first attempt to direct that youth to a provider that ultimately county Behavioral Health authorities would deem inappropriate. Agreed-upon options for provider destinations decreased significantly after tackling these questions at the local Pathway Meetings, allowing for more clarity and consistency.

The relatively low overlap of appropriate service destinations across agencies and availability of multiple options for care (not all of which would be agreed-upon **best** options) would likely have resulted in confusion regarding the best ways to manage youth in crisis among juvenile justice staff. Managing youth suicide risk is likely very stressful for justice staff, who are not trained as clinicians. Clarifying and simplifying handoff procedures to connect the young persons under their supervision to behavioral health care would be a positive outcome, anticipated to both increase the likelihood of service access for those youths and to lower staff stress. In earlier work, we tracked steps in Behavioral Health service access for over 8300 youths undergoing intake into community juvenile justice agencies in seven states (Wasserman et al., 2021). In that work, even when youths’ service needs were clearly identified and documented via screening or other means by the juvenile justice agency, only 21.9% of youths identified as in need of Behavioral Health services were actually referred on to such providers. While both family and system barriers play well-documented roles in the low rates of Behavioral Health service access for justice-involved youths, the work presented here suggests that another contributor to lack of needed referral may be agency confusion about the best and most efficient option(s) for care (Elkington et al., 2020).

The importance of the Pathway Meetings in clarifying and structuring the agreed upon Pathways was a key part of the process. While the meetings began with clear goals and format, the structure of the resulting Pathways at the outset of each meeting was unknown. The meetings were organized via the overarching steps of the Behavioral Health cascade, with a focus on determining specific actions to be taken with each step, beginning after a youth was screened. The final Pathways, which emerged following an iterative process of review and approval between the academic research team and the Behavioral Health/justice system representatives after the Pathway Meeting, all had a similar structure. Regardless of the number of steps in a given Pathway, all had steps that centered on *Debriefing* (e.g. discussing with the family the screening results), *Connecting* the youth to treatment, in which the relevant Behavioral Health provider is alerted and screening results are shared, and then *Documenting* events and results in the Probation information management system and informing an existing provider if this person was not alerted earlier (i.e., during *Connect* steps). Thus, the meetings attended by both Behavioral Health and Juvenile Probation systems provided an opportunity for these county partners to contribute direction on how the notification process between Probation and the family and between the two agencies should work, and which agency or (emergency) provider should be assigned to each risk classification.

Importantly, the act of bringing Behavioral Health *and* Juvenile Probation systems together to address and make joint decisions regarding the referral Pathways for each class of youth resulted in novel and innovative solutions. Some useful options had not been considered by either system on its own prior to meetings but were embraced in discussions guided by the external facilitator (academic partner). For example, neither system identified a Mobile Crisis team as a viable response option to Class I or II in any county; in Probation settings typically 911/law enforcement would be asked to fill that role. Yet, with the integrated approach to determining a responsive Pathway for a clearly defined category of youth, the result was a utilization of appropriate services and an expansion of the behavioral health system within a given county.

While few investigations have examined their effectiveness, best practices recommendations commonly include building interagency bridges through the use of *Memoranda of Understanding* (or Memoranda of Agreement: Taxman & Bouffard, 2000; Fletcher et al., 2009; Lehman et al., 2009). For such work, MOUs can establish goals, identify procedures, and solidify leadership support. On the other hand, the key to success is not always at the top of the organization but often consists of attention to the novel work processes where interagency efforts must be reconfigured across organizational boundaries; these efforts involve commitment from front-line staff and reallocation of resources (Taxman & Bouffard, 2000). The MOU represents an agency commitment but does not guarantee that systems can be built or that the critical pieces can be addressed. *Boundary spanners*, or personnel who have commitments to more than one organization, are also recognized as front-line facilitators of this type of refined work processes. The boundary spanner generally reports to both agencies, working across organizational lines. Due to limited resources, these positions are rare, and again there has been little evaluation of their effectiveness as facilitators.

As another alternative, the *local change team* evolved as a means for working through the organizational issues related to redesigning work processes. Change teams have demonstrated effectiveness in programs implementing HIV testing in prisons (Belenko et al., 2013), increasing comfort with medication assisted treatments for substance use disorders among Probation and health staff (Welsh et al., 2016), and implementing revised case management practices in justice settings (Magnuson et al., 2020). This study presents another use of local change team to bridge service systems.

External facilitators (including technical assistance providers, experts in the field, and academic-research partnerships) can further assist change teams to achieve their goals and address barriers to change (Berta et al., 2015; Ford et al., 2007; Harvey et al., 2018; Lesard et al., 2015; Magnuson et al., 2020; Ritchie et al., 2019). Because the external facilitator can be seen as impartial and driven by the practice change goal, s/he is well-positioned to negotiate differences and to work towards inter-agency consensus decisions.

This study illustrated the value of relying on interdisciplinary local change teams coupled with an academic partnership to forward the development of work processes that accommodate planned rollout of e-Connect system protocols and use of clinical guidelines to address youths at risk for suicidal behavior. Both bridging and reliance on academic partners are key factors in the EPIS implementation framework, and the present investigation demonstrates the value of integrating both into the planning process. The academic partner brings knowledge of the research literature, ability to design and implement evaluation research, flexibility in dealing with different contexts, and provides an independent perspective (Rudes et al., 2014). Here, the academic partner provided a variety of types of assistance to help the interdisciplinary teams work through difficult issues, particularly around the necessity of Probation addressing the risks related to suicidal behavior in Probationers, and the linkage to service providers. Columbia University’s team consisted of well-respected clinical researchers who attended team meetings and were available to provide the local change teams with expertise in suicide risk and management in adolescents, systems processes, and screening and assessment practices. This expertise helped to guide each local team to undergo the planning and develop procedures that would allow Juvenile Justice and Behavioral Health agencies to proceed with implementing e-Connect protocols. Finally, because of their grounding in both service sectors and their experience (both research and implementation) with screening and assessment, the academic partnership provided the credibility needed to promote agreement to a clinical Pathways framework sensitive to the needs of both agencies and their staff, as well as to youths and their families.

Many factors, at family and system levels, likely contribute to the often-reported low rates of behavioral health service access for justice system youths. We continue to struggle with best approaches to break down these barriers, with programs to motivate families and individuals and with initiatives targeting both service delivery and costs (Ivey-Stephenson et al., 2020). We need also to direct effort toward better planning aimed at efficiency in cross-agency service linkage: agency policies may be more receptive to such change than other targets. While the efforts described here resulted in more efficient Pathways, future work will examine the degree to which the processes laid out in e-Connect are successful in linking justice youths to needed community services.

## Conclusions

Increasingly, community justice agencies are incorporating evidence based behavioral health screening into their routine practice, as a means to best direct youths to needed services and to appropriately divert them from further justice system contact (King et al., 2000; Scott et al., 2019). While laudable from many perspectives, this practice has left justice agencies with a need to better manage youth triage to other sectors of care. Without advance planning, when such screening identifies youth in crisis, non-clinician staff in justice agencies are likely to experience stress that often complicates decision-making (Levitin, 2014). Advance planning that establishes and routinizes the most acceptable paths to care for youths at risk for suicidal behavior is an important component in the necessary inter-agency collaboration that such planning requires. The planning process for such critical management events can be well-supported by the activities of an engaged academic partnership.

## Data Availability

The coding data and analysis are available from the corresponding author on reasonable request.

## Abbreviations

EPIS: The Exploration, Preparation, Implementation, Sustainment framework (a common implementation science framework
EBPs: Evidence-based practices
MOU: Memoranda of Understanding
SB: Suicidal behavior
